# Editorial: The Potential Effect and Mechanism of Traditional Medicine on Vascular Homeostasis and Remodeling: An Update

**DOI:** 10.3389/fphar.2022.847333

**Published:** 2022-03-10

**Authors:** Yuefan Zhang, Tie-Jun Li, Mengting Lv, Xinyu Li, Yuliang Wang

**Affiliations:** ^1^ School of Medicine, Shanghai University, Shanghai, China; ^2^ Plant Biotechnology Research Center, Joint International Research Laboratory of Metabolic and Developmental Sciences, Fudan-SJTU-Nottingham Plant Biotechnology R&D Center, School of Agriculture and Biology, Shanghai Jiao Tong University, Shanghai, China; ^3^ Key Laboratory of Urban Agriculture (South) Ministry of Agriculture, Plant Biotechnology Research Center, Fudan-SJTU-Nottingham Plant Biotechnology R&D Center, School of Agriculture and Biology, Shanghai Jiao Tong University, Shanghai, China

**Keywords:** vascular homeostasis, vascular remodeling, nature product, cardiovascular, Chinese traditional and herbal drugs

Cardiovascular diseases are the leading causes of death worldwide. And most cardiovascular diseases are accompanied by vascular homeostasis disorder and vascular remodeling. Vascular remodeling can disrupt the homeostasis of blood vessels, lead to vascular dysfunction and induce organ injury. Moreover, vascular remodeling is also the pathological basis of cardiovascular diseases and metabolic diseases such as hypertension, coronary heart disease, stroke, heart failure, diabetes, atherosclerosis (Koenen et al., 2021). In recent years, a growing body of experimental studies on the regulation of vascular remodeling and vascular homeostasis by natural products, which will lay the foundation for new drug discovery in cerebrovascular diseases.

Following the successful “The Potential Effect and Mechanism of Traditional Medicine on Vascular Homeostasis and Remodeling” Research Topic in *Frontiers in Pharmacology*, we organized a new Research Topic entitled “*The Potential Effect and Mechanism of Traditional Medicine on Vascular Homeostasis and Remodeling: An Update*” in *Frontiers in Pharmacology*. A total of nine articles has been published on this Research Topic, including eight research articles and one review. Here, we summarize the highlights of these articles.

Small vessel diseases are the disorders of cerebral microvessels that causes stroke and vascular dementia, which lead to white matter damage in cardiovascular diseases. The cerebral microvessel lesions manifest as blood-brain barrier dysfunction, impaired vasodilation, vessel stiffening, white matter rarefaction, and cerebral ischemia (Wardlaw et al., 2019). Many natural products have therapeutic effects on small vessel diseases. Quercetin is a flavonoid found in many plants. (Li M. et al, 2021).show that Quercetin mitigates HR-induced human brain microvascular endothelial cell injury by promoting the viability, migration and angiogenesis of HBMECs (Human Brain Microvascular Endothelial Cells), and inhibiting the apoptosis of HBMECs. Liquiritin is the main active component of *Glycyrrhiza uralensis Fisch*. The research article by (Li MT. et al, 2021)is focused on the protective effects of liquiritin on HR-induced human brain microvascular endothelial cells. The authors found that Liquiritin promotes proliferation, migration, angiogenesis, and inhibits the apoptosis of HBMECs. Quercetin and Liquiritin might serve as protective compounds in small vessel diseases by improving blood-brain barrier.

The disruption of vascular homeostasis is closely related to the dysfunction of endothelial cells and smooth muscle cells in the blood vessels. Regulating the function of intravascular cells can effectively improve vascular remodeling and vascular homeostatic imbalance. Atherosclerosis is characterized by the dysfunction of lipid and vascular homeostasis. Chen et al. explore the therapeutic effects of Tongxinluo, a traditional Chinese medicine, on atherosclerosis, and find Tongxinluo can attenuate the accumulation of lipids, improve fat metabolism in macrophage. This effect relies on enhancing Beclin-1-induced autophagy. Resibufogenin is an active compound from *Bufo bufonis*. A study by Yang et al. shows Resibufogenin exerts the antiangiogenic effect on the HUVECs (Human Umbilical Endothelial Cells) in a VEGFR2 (Vascular Endothelial Growth Factor 2) dependent signal pathway.

Hu-zhang-qing-mai-yin is a Chinese traditional medicine formula, has been clinically used for many years. The research article by Yu et al. is focused on the anti-diabetic retinopathy effects of Hu-zhang-qing-mai-yin *in vitro*. They show Hu-zhang-qing-mai-yin can inhibit the cell proliferation and promote the mitochondria-related apoptosis of human retinal capillary endothelial cells through P38 and NF-κB signaling pathways. Avenanthramide C is a component of the phenolic compound of oats. Park et al. show Avenanthramide C can inhibit the expression of MMP (Matrix Metalloproteinase) and migration through MAPK (Mitogen-Activated Protein Kinase) signaling in human aortic smooth muscle cells. Avenanthramide C could be a promising candidate for atherosclerosis diseases.

**FIGURE 1:**
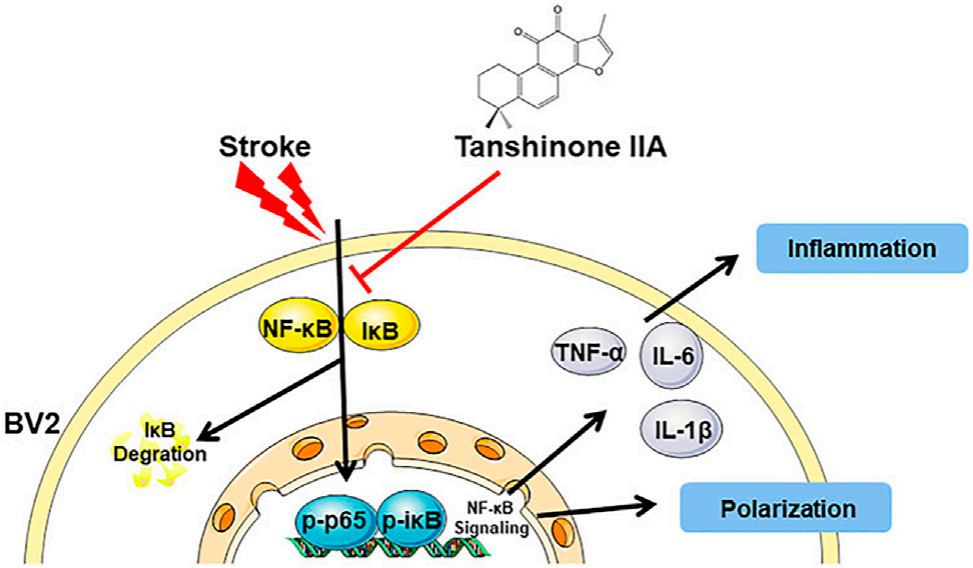
The mechanism of the neuroprotective mechanisms of Tanshinone IIA in stroke-induced neuroinflammation.

Stroke is the sudden rupture of blood vessels in the brain, or the blockage of blood vessels in the brain, resulting in a series of brain damage. Now it is the main cause of death and disability worldwide. And the stroke burden is a huge public health issue (Owolabi et al., 2021). Drug research for stroke prevention and treatment has been a hot topic in medical research. A series of articles within this Research Topic identify novel natural products that can alleviate the cerebral ischemia injury. MQ (L-methionyl-L-glutamic acid) is one of the metabolites of monocyte locomotion inhibitory factor, a thermostable pentapeptide secreted by *Entamoeba histolytica*. The work by Zhang et al. describes the role of MQ in cerebral ischemia. They found that MQ exerts a neuroprotective effect in cerebral ischemia by blocking apoptosis via the p-JNK/Bax pathway. Tanshinone IIA is a fat-soluble diterpenoid isolated from *Salvia miltiorrhiza Bunge*. Song et al. show that Tanshinone IIA also has a neuroprotective effect by regulating microglia polarization from M1 to M2 in an NF-κB dependent signaling pathway (Figure 1). The review by Kang et al. brings the information regarding the protective effect and mechanism of astragaloside IV, an extract from *Radix Astragali*. Astragaloside IV exerts a protective effect on ischemia-reperfusion injury by relieving neuronal apoptosis, oxidative stress, BBB injury, leukocyte adhesion, and inhibiting inflammatory reaction.

Vascular remodeling is the pathological basis of vascular and circulatory dysfunction. The process of vascular remodeling is complex, which is closely related to abnormal hemodynamics, activation of vasoactive substances, cell proliferation and apoptosis, inflammation and oxidative stress. Natural products play an important role in regulating vascular remodeling and promoting vascular homeostasis because of their multi-target properties. Although articles presented in this Research Topic illustrate natural products that regulate vascular remodeling and vascular homeostasis, many natural products that regulate vascular function and the role of natural products in vascular remodeling and vascular homeostasis have not been clarified. We hope we can bring an update Research Topic soon.
